# Method of Administering Quinine

**Published:** 1847-09

**Authors:** 


*Method of administering Quinine.*—An infusion of coffee is said to conceal the bitterness of quinine without impairing its medicinal virtues, and it is recommended to give the medicine in such an infusion.—*Ibid.*

